# Immunohistochemical characterization of neoplastic cells of breast origin

**DOI:** 10.1186/1746-1596-7-73

**Published:** 2012-06-22

**Authors:** MariadelasMercedes Noriega, Fernando Paesani, Florencia Perazzo, Néstor Lago, Hugo Krupitzki, Silvana Nieto, Alejandro Garcia, Alejandra Avagnina, Boris Elsner, Valeria Cecilia Denninghoff

**Affiliations:** 1 Centro de Educación Medica e Investigaciones Clínicas “Norberto Quirno” (CEMIC), Ciudad Autónoma de Buenos Aires, Argentina; 2 GEMATECH, Ciudad Autónoma de Buenos Aires, Argentina; 3 Department of Pathology, CEMIC, Galván 4102, 1st floor. C1431FWO, Ciudad Autónoma de Buenos Aires, Argentina

**Keywords:** Tumors of unknown origin, Breast, Mammaglobin, Immunohistochemistry

## Abstract

**Background:**

After skin cancer, breast cancer is the most common malignancy in women. Tumors of unknown origin account for 5-15% of malignant neoplasms, with 1.5% being breast cancer. An immunohistochemical panel with conventional and newer markers, such as mammaglobin, was selected for the detection of neoplastic cells of breast origin. The specific objectives are: 1) to determine the sensitivity and specificity of the panel, with a special emphasis on the inclusion of the mammaglobin marker, and 2) to compare immunohistochemistry performed on whole tissue sections and on Tissue Micro-Array.

**Methods:**

Twenty-nine metastatic breast tumors were included and assumed as tumors of unknown origin. Other 48 biopsies of diverse tissues were selected and assumed as negative controls. Tissue Micro-Array was performed. Immunohistochemistry for mammaglobin, gross cystic disease fluid protein-15, estrogen receptor, progesterone receptor and cytokeratin 7 was done.

**Results:**

Mammaglobin positive staining was observed in 10/29 cases, in 13/29 cases for gross cystic disease fluid protein-15, in 20/29 cases for estrogen receptor, in 9/29 cases for progesterone receptor, and in 25/29 cases for cytokeratin 7. Among the negative controls, mammaglobin was positive in 2/48, and gross cystic disease fluid protein-15 in 4/48.

**Conclusions:**

The inclusion of MAG antibody in the immunohistochemical panel for the detection of tumors of unknown origin contributed to the detection of metastasis of breast cancer. The diagnostic strategy with the highest positive predictive value (88%) included hormone receptors and mammaglobin in serial manner.

**Virtual slides:**

The virtual slide(s) for this article can be found here: http://www.diagnosticpathology.diagnomx.eu/vs/1366310812718988

## Background

After skin cancer, breast cancer is the most common malignancy in women, accounting for 16% of all cancers in women [1]. Tumors of unknown origin represent a heterogeneous group of tumors that first manifest as metastasis in a patient with unknown history of disease, where the assessment fails to identify the primary tumor site at diagnosis. Tumors of unknown origin account for 5 to 15% of all malignant neoplasms [2,3]. Breast cancer accounts for only 1.5% of tumors of unknown origin. Therefore, it is one of the first tumors to be ruled out in the presence of tumors of unknown origin in the female population. An adequate diagnosis has an impact on the treatment and prognosis of the disease [4]. Immunohistochemistry is a diagnostic tool for the classification of tumors of unknown origin, but few organ-specific markers allow to accurately determine the origin of a metastasis, and the use of an immunohistochemical algorithm requires efficient and fast immunohistochemical testing [5]. To date, several breast markers have been postulated, such as beta-catenin, FAK, PIP, MUC1, PSE, e-cadherin, cytokeratin 7 (CK7), estrogen and progesterone receptors (ER and PR), gross cystic disease fluid protein-15 (GCDFP-15), mammaglobin (MAG)-A and MAG-B [6-9]. Fritzsche *et al.* showed 73.3% and 72.1% cytoplasmic positivity for GCDFP-15 and MAG, respectively, in invasive breast cancer (primary tumor) [10]. These findings significantly correlated with low tumor grade. Chia *et al.* demonstrated 47.8% and 11.3% positivity for MAG and GCDFP-15, respectively, in neoplastic cells of breast origin [11]. On the other hand, Takeda *et al.* found a sensitivity of 45% and 50% for GCDFP-15 and MAG, respectively, in pulmonary and pleural metastasis from breast cancer [12]. Moreover, Bhargava *et al.* showed positivity in 55.4% and 23.1% for MAG and GCDFP-15, respectively, in breast carcinomas as primary tumors [13]. None of them is specific for this type of neoplasm, and they all express uneven positivity in other malignancies. In addition, their sensitivity for breast cancer is variable, hence the need to use them as panels and correlate the results with the morphology of the tumor. Used alone, MAG-A could be the marker of choice to identify a tumor of breast origin [14]. According to these findings, this novel antibody may be included in an immunohistochemical panel. Antibodies used for the diagnosis of breast carcinomas were analyzed. To our knowledge, none of them is sensitive and specific enough to be used alone. Therefore, there is no Gold Standard available for breast carcinoma. The aim of this study was to determine the specificity and sensitivity of both the antibodies alone and the panel as a whole, using a panel of immunohistochemistry: CK7, hormone receptors (estrogen and progesterone), GCDFP-15 and MAG. We hypothesize that the inclusion of the MAG antibody to the immunohistochemical panel already routinely used would increase the positive predictive value in the detection of breast tumors of unknown origin.

## Methods

The population consisted of 77 patients (29 cases and 48 negative controls). Twenty-nine cases were selected and followed at CEMIC Laboratory of Pathology. Samples were collected from both the primary breast tumor and its metastases at different anatomical sites. The 29 metastases were considered as tumors of unknown origin, despite the known origin of the breast tumor. Furthermore, another 48 biopsies were selected, including 42 different samples of primary or metastatic non-mammary tumors, and 6 non-neoplastic tissue biopsies. These samples were used as immunohistochemical negative controls, with all the possible sites of breast tumor metastasis included.

### **Immunohistochemistry**

Four- to five-micron tissue sections were done and stained with hematoxylin-eosin, whereas 3-4-μm tissue sections (paraffin blocks and Tissue Micro-Array) were used for immunohistochemistry. The following monoclonal antibodies were used: MAG clone 304-1A5 (DAKO, Carpinteria, CA 93013, USA), CK7 clone OV-TL 12/30 (Dako, Carpinteria, CA 93013, USA), ER clone 1D5 (DAKO, Carpinteria, CA 93013, USA), PR clone 16 (Novocastra Laboratories Ltd. Balliol Business Park West, Benton Lane, Newcastle Upon Tyne NE128EW United Kingdom), and GCDFP-15 clone 23 A 3 (DBS 1020 Serpentine Lane, FF114, Pleanton, CA 94566).

### **Tissue micro-array**

A technique known as Tissue Micro-Array facilitates the simultaneous staining of many specimens on the same slide. It is the orderly distribution of hundreds of tissue samples in a tiny cylindrical paraffin block. Thus, since 1998, a large body of scientific evidence on Tissue Micro-Array has been reported, where this practical technique has been widely used. A blank paraffin-block was selected, which was punched at preset coordinates using Manual Tissue Arrayer I (Beecher Instruments), and 0.6 mm needles. At these sites, tumor samples drawn from the original blocks were inserted in turn. Three samples were punched from each tumor. Thus, the assembled block comprised a triplicate of each case.

### **Statistical analysis**

Five different strategies were developed in order to define the diagnosis of metastatic breast cancer. For each strategy, sensitivity, specificity and positive predictive value were determined. The first two strategies were performed in parallel. The first one (strategy 1) considered as positive all those cases with hormone receptors (ER and PR), CK7 and GCDFP-15. The second one (strategy 2) included the same aforementioned panel, plus MAG. The remaining three (strategies 3, 4 and 5) were considered in series, and they first analyzed hormone receptors (ER and PR). In those cases where hormone receptors were negative, either CK7 or MAG was added (strategies 3 and 4). In this sense, a strategy model was developed, basically considering ER and/or PR in the first place, followed by MAG and then CK7 (strategy 5).

## Results

Positive staining is shown in Table 1. Of 10/29 positive metastatic breast carcinomas, 4 were invasive ductal carcinoma (IDC), 2 invasive lobular carcinoma, 3 were diagnosed as carcinomas of breast origin (not otherwise specified, NOS), and 1 was undifferentiated carcinoma. The sites of the metastases where MAG stained positively included brain, lymph node, pleura, ovary, bone and bone marrow. The immunoreactivity pattern ranged between focal and diffuse staining, with varying intensities.

**Table 1:** Positive immunohistochemical results

**Marker**	**Cases (n = 29)**	**Negative controls (n = 48)**
MAG	10/29 (34%)	2/48 (4%)
GCDFP-15	13/29 (45%)	4/48 (8%)
ER	20/29 (69%)	0/48 (0%)
PR	9/29 (31%)	0/48 (0%)
CK7	25/29 (86%)	18/48 (38%)

***MAG*:** mammaglobin; ***GCDFP-15*:** gross cystic disease fluid protein; *E****R*:** estrogen receptors; ***PR*:** progesterone receptor; ***CK7*:** cytokeratin.

On the other hand, the sites of the 13/29 metastases positive for GCDFP-15 were lymph node, brain, ovary, bone, bone marrow and pleura. The type of staining observed was similar to that of MAG.

In the cross analysis of antibodies, 8/29 cases were positive for both MAG and GCDFP-15. All cases positive for PR were also positive for ER. Statistics is shown in Tables 2 and 3.

**Table 2:** Statistical results for each marker

**Marker**	**TP**	**TN**	**FP**	**FN**	**S (%)**	**Sp (%)**	**PPV (%)**	**+LR**	**−LR**
MAG	10	40	2	19	34	95	83	7.24	0.69
GCDFP-15	13	38	4	16	45	90	76	4.71	0.61
HR	20	41	1	9	69	98	95	29.0	0.32
CK7	25	23	19	4	86	55	57	1.91	0.25

***MAG:*** mammaglobin; ***GCDFP-15*:** gross cystic disease fluid protein; ***HR:*** estrogen and progesterone receptors; ***CK7:*** cytokeratin 7; ***TP:*** true positives; ***TN:*** true negatives; ***FP:*** false positive; ***FN:*** false negative; ***S:*** sensitivity; ***Sp:*** specificity; ***PPV:*** positive predictive value; ***+ LR***: positive likelihood ratio; **− LR**: negative likelihood ratio.

**Table 3:** Immunohistochemical strategies, statistical results

	**Strategy**	**TP**	**TN**	**FP**	**FN**	**S (%)**	**Sp (%)**	**PPV (%)**	**+LR**	**−LR**
	**Markers considered in a parallel manner**									
1	HR + CK7 + P-15	10	42	0	19	35	100	100	999.999	0.66
2	HR + CK7 + P-15 + MAG	7	42	0	22	24	100	100	999.999	0.76
	**Markers considered in a serial manner**									
3	HR----CK7	28	20	22	1	97	48	56	1.84	0.07
4	**HR----MAG**	21	39	3	9	70	93	**88**	9.80	0.32
5	HR----MAG----CK7	28	22	20	1	97	52	58	2.03	0.07

**HR:** estrogen and progesterone receptors; **CK7:** cytokeratin 7; **P-15**: gross cystic disease fluid protein (GCDFP-15); **MAG:** mammaglobin; **TP:** true positives; **TN:** true negatives; **FP:** false positive; **FN:** false negative; **S:** sensitivity; **Sp:** specificity; **PPV:** positive predictive value; **+ LR**: positive likelihood ratio; **− LR**: negative likelihood ratio.

Immunohistochemistry of tissue Micro-Array and complete sections of the tumor were compared, and no significant differences were observed, except for 2 cases of metastasis of breast origin. In one of them, staining with GCDFP-15 was not observed in the field punched for the Tissue Micro-Array. The same occurred with MAG antibody in the other case.

## Discussion

For tumor diagnosis, primary location, morphology, histology and cytology, as well as clinical data, are crucial. According to the literature, the sensitivity and specificity of MAG and GCDFP-15 for the detection of breast cancer vary significantly, with MAG sensitivity being 80% to 48%, and that of GCDFP-15 being 73% to 23% [9,13,15]. In our study, MAG sensitivity was 34%, and its specificity 83%, while the sensitivity and specificity of GCDFP-15 were 44% and 79%, respectively. While MAG sensitivity is lower than that of GCDFP-15, its positive predictive value is higher, being 83% for MAG and 76% for GCDFP-15. This shows that the inclusion of MAG antibody in the immunohistochemical panel would contribute to the detection of metastasis of breast cancer. According to the literature, CK7 antibody was positive in 25/29 cases (86%), which reveals an 83% positivity in breast cancer [16].

Immunomarkers in the negative control group revealed that: 1) MAG and GCDFP-15 were both positive in a case of pancreatic adenocarcinoma and another of cholangiocarcinoma. Review of the literature reports 10% of cholangiocarcinomas positive for MAG, while other source reveals MAG expression in intestinal tumors, mainly adenocarcinomas [17]; 2) GCDFP-15 showed positivity in a case of colorectal adenocarcinoma and another of large cell lung carcinoma. In these cases, the lack of MAG staining contributes to a higher positive predictive value, with more chances to detect metastasis of breast carcinoma. This shows that the inclusion of MAG in the immunohistochemical panel is highly convenient. 3) CK7 was positive in 20/48 of the negative controls included, which were mainly adenocarcinomas (12/48); 4) MAG is usually found in the female genital tract cells, and has been expressed in up to 77% of endometrial carcinomas (as primary tumor) [18,19]. However, in our study, female genital tract cells were not positive for this antibody.

As shown in Table 3, the addition of MAG to a basic immunohistochemical panel (ER. PR, CK7 and GCDFP-15) is associated with lower sensitivity. The best immunohistochemical strategy seems to be number 4, which considers hormone receptors and the inclusion of MAG in a serial manner. Despite the low sensitivity of this diagnostic approach to metastatic carcinoma of breast origin, it offers high specificity (Table 3). As far as probability, the use of the immunohistochemical panel herein proposed proved categorical in only three cases, but allowed the exclusion of all the controls.

Jae-Ho *et al.* proposed the inclusion of MAG within the immunohistochemical panel for the detection of metastatic breast cancer [20]. Yang *et al.* concluded that MAG would be a good marker to be included in an immunohistochemical panel for differentiation between lung tumors and metastatic breast cancer in the lung [21]. MAG sensitivity was lower than that of GCDFP-15, but had a higher specificity. When combined, the whole sensitivity decreased, but an increase in specificity was observed, with no decrease in the positive predictive value. Overall, the use of the immunohistochemical panel contributes to the detection of metastasis of breast origin. Laurinavicius *et al.* showed the benefit of integrating digital image analysis into routine pathology, in order to determine breast ductal carcinoma immunohistochemical profiles by using multiple immunohistochemical biomarkers. Thus, the potential inclusion of MAG in these panels, as a tumors of unknown origin marker, would be promising for the diagnosis and treatment of these patients in the future [22,23].

## Conclusions

The inclusion of MAG antibody in the immunohistochemical panel for the detection of tumors of unknown origin contributed to the detection of metastasis of breast cancer. The diagnostic strategy with the highest positive predictive value (88%) included hormone receptors and mammaglobin in serial manner.

## Abbreviations

CK7: CytoKeratin 7; ER: Estrogen Receptor; FN: False Negative; FP: False Positive; MAG: Mammaglobin; GCDFP-15: Gross Cystic Disease Fluid Protein; PR: Progesterone Receptor; S: Sensitivity; Sp: Specificity.

## Competing interest

All the authors declare that they have no competing interest in this protocol.

## Authors' contributions

All the authors of this protocol have made a substantial contribution to its conception and design, data collection, analysis and interpretation.
